# Clinical Utility of BRAF, NRAS, and TERT Promoter Mutation in Preoperative Thyroid Fine-Needle Aspiration Biopsy: A Diagnostic Study From Dharmais Cancer Hospital

**DOI:** 10.31557/APJCP.2020.21.11.3267

**Published:** 2020-11

**Authors:** Adhitya Bayu Perdana, Rizky Ifandriani Putri, Rachmawati Rachmawati, Bob Andinata, Bayu Brahma

**Affiliations:** 1 *Department of Research and Development, Dharmais Cancer Hospital - National Cancer Center, Jakarta, Indonesia. *; 2 *Department of Anatomical Pathology, Dharmais Cancer Hospital - National Cancer Center, Jakarta, Indonesia. *; 3 *Surgical Oncology Study Program, Department of Surgery, Faculty of Medicine Universitas Indonesia - Cipto Mangunkusumo Hospital, Jakarta, Indonesia. *; 4 *Department of Surgical Oncology, Dharmais Cancer Hospital - National Cancer Center, Jakarta, Indonesia. *

**Keywords:** Fine-needle aspiration biopsy, hotspot mutation, thyroid cancer, diagnostic

## Abstract

**Objective:**

Molecular testing of thyroid nodules becomes important for improving the accuracy of fine-needle aspiration biopsy (FNAB). This study aimed to investigate the diagnostic utility of *BRAF*, *NRAS*, and *TERT* promoter mutation in thyroid nodules at Dharmais Cancer Hospital.

**Methods:**

We performed a prospective diagnostic study involving 50 patients with thyroid nodules who needed surgery between September 2013 and August 2014. Mutational hotspots in *BRAF* exon 15, *NRAS* exon 3, and *TERT* promoter region were analyzed by Sanger sequencing from FNAB specimens. Cytology and molecular data were compared to histopathology results.

**Results:**

Of the 50 cases included in the analysis, 39 cases (78%) were thyroid malignancies. Mutations of *BRAF*, *NRAS*, and *TERT* promoter were detected in 31% (12/39), 18% (7/39), and 13% (5/39) cases, respectively. *BRAF* and *NRAS* mutations were found mutually exclusive, while all of *TERT* promoter mutation was found coexistent either with *BRAF* (40%) or *NRAS* (60%). The combination of FNAB cytology and molecular testing resulted in 69% sensitivity, 100% specificity, 100% positive predictive value, 48% negative predictive value, and 76% accuracy.

**Conclusion:**

Molecular testing of *BRAF*, *NRAS*, and *TERT* mutations improve the sensitivity of thyroid FNAB and is beneficial for more definitive treatment in selective cases. However, the NPV is relatively low to avoid the need for diagnostic surgery. Therefore, further studies to identify more sensitive methods and more comprehensive molecular markers in the diagnosis of thyroid nodules are needed.

## Introduction

The incidence of thyroid cancer has surged globally in the past three decades, with papillary thyroid carcinoma (PTC) as the most frequent histopathologic subtypes (Bray et al., 2018; Liu et al., 2017; Pellegriti et al., 2013). According to GLOBOCAN data in 2018, there were 11,470 new cases and 2,119 deaths of thyroid cancer in Indonesia (World Health Organization, 2019). Although the mortality rate of thyroid cancer is only 0.4% of all cancer deaths, the identification for malignancy of thyroid gland enlargement remains a problem considering many non-cancer diseases of thyroid nodules (Kartini and Wibisana, 2017).

Fine needle aspiration biopsy (FNAB) has been broadly accepted as an initial screening test for patients with thyroid nodules. This test has led to a better assessment for patient’s treatment decisions with a high sensitivity rate reported from 65% to 98%, respectively (Gharib and Goellner, 1993; Yang et al., 2007). However, about 10-40% of cases are diagnosed as indeterminate for malignancy and up to 10% of benign cytology may have a false-negative result (Nikiforov et al., 2009; Yeh et al., 2004). These findings demand improvement for diagnostic accuracy of patients with indeterminate cytology results.

Recent molecular markers in thyroid cancer have been applied to reinforce the FNAB examination since 60-70% of malignancy harbor at least one known genetic alteration (Alexander et al., 2012; Nikiforov et al., 2009). Based on previous reports, there are currently several biomarkers that have clinical implications for thyroid cancer including RET/PTC, RAS, *BRAF*, PAX8-PPARγ, MicroRNAs (miR-221, 222, and 181b), and activation of telomerase reverse transcriptase (*TERT*) (Albarel et al., 2012; Liu et al., 2012; Moses et al., 2010; Nikiforov et al., 2009; Pallante et al., 2006). However, in terms of diagnostic utility, *BRAF*, *NRAS*, and *TERT* promoter mutations have shown greater potential due to the high prevalence and clinical features in thyroid cancer (Liu et al., 2012; Moses et al., 2010; Nikiforov et al., 2009).

*BRAF* mutation is the most frequent event in thyroid cancer, especially in PTC classic variant subtype (Shibru et al., 2008; Xing, 2005; Xu et al., 2003). The majority of *BRAF* mutations occur in exon 15 at codon 600 (V600E) and lead to stimulation of the MAPK signal transduction pathway, resulting in increased cell proliferation (Albarel et al., 2012; Xing, 2005). Various studies have demonstrated that *BRAF* V600E mutation testing has high specificity and is useful for the clinical diagnosis of PTC in indeterminate FNAB specimens (Brahma et al., 2013; Cohen et al., 2004; Jin et al., 2006; Rowe et al., 2006; Su et al., 2016).

The second most common genetic alteration in thyroid cancer is RAS mutations. Despite the diagnostic importance of RAS mutations that is still not fully clear as these mutations are also found in benign nodules, it is highly predictive in tumors of follicular cell origin, which are difficult to differentiate on cytology (Alexander et al., 2012). RAS mutations were found about 93% in indeterminate cytology, with the vast majority of RAS-positive tumors, which are PTCfv (Gupta et al., 2013). Among RAS family members, *NRAS* codon 61 (Q61R) is the most frequent mutation accounted for 67-88% of all RAS mutations (Bae et al., 2014; Nikiforov and Nikiforova, 2011).

The recently identified *TERT* promoter mutations are also recognized as a clinically important diagnostic marker for thyroid cancer (Argyropoulou et al., 2018). Two promoter mutations, 228C>T and 250C>T in *TERT* gene have shown a significant role in the pathogenesis of thyroid cancer, with 228C>T as the most frequently occurring compared to the 250C>T (Liu and Xing, 2014, 2016). The high prevalence of *TERT* promoter mutations is found in aggressive thyroid tumors, showing high specificity for malignant neoplasm (Kim et al., 2016). Moreover, *TERT* promoter mutation-positive was also found in several cases of indeterminate results. Thus, it has great potential for a definitive preoperative diagnosis of thyroid nodules (Liu and Xing, 2014).

Considering the diagnostic accuracy improvement in cytology specimens, the synergism of these three biomarkers should be underlined. To our knowledge, only a few studies have reported the combination of these mutations in the preoperative diagnostic study. Besides, the report for multiple hotspot mutation analyses of thyroid cancer in Indonesia is not available yet. Therefore, we aimed to investigate the diagnostic utility of *BRAF*, *NRAS*, *TERT* promoter mutation, and its combination from our FNAB specimen series at Dharmais Cancer Hospital.

## Materials and Methods

*Study participant*

**Figure 1:**
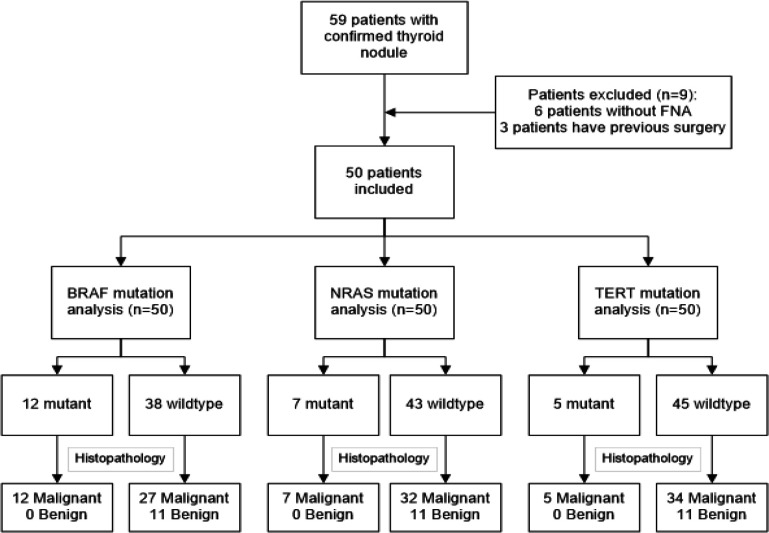
Patients Inclusion Flowchart for Mutation Analysis

A total of 50 FNAB specimens were prospectively collected from patients treated at Dharmais Cancer Hospital between September 2013 and August 2014. The inclusion criteria were new patients with thyroid nodules and who could undergo surgery. The patients with other malignancies, or had a history of thyroid surgery with histopathology results, and refused to participate in the study were excluded from this study. The inclusion flowchart can be seen in Figure 1. Patient’s characteristics including age, sex, the onset of disease, tumor size, lymph node metastasis, tumor stage, cytology, and histopathology results were obtained from the patient’s medical record.

This study was approved by the Research Ethics Committee of Dharmais Cancer Hospital with the registration number 039/KEPK/XI/2013, and informed consent was obtained from all of the patients included in this study. All data were anonymized before analysis.

*FNAB cytology and histopathology review*

The retrieval of FNAB cytology specimens was carried out preoperatively by a pathologist (RIP). If the patients refused for preoperative FNAB, then the intraoperative procedure was performed by a surgical oncologist (BB). For the preoperative procedure, the 24-gauge needle was inserted and monitored during the biopsy procedure. The aspirated liquid was partially daubed on a glass object and fixed with 95% alcohol for Papanicolaou smear, and then examined under a microscope. The remaining FNAB specimens in the needle were washed out by phosphate-buffered saline (PBS) solution 1x and inserted in a 2-ml collection tube. For intraoperative procedures in the operating theatre, the aspiration of the nodule was carried out after the removal of the thyroid gland from the patients. All FNAB specimens were stored in 4oC before the DNA extraction procedure. The diagnosis categories for cytology were benign, atypia of undetermined significance (AUS)/follicular lesion of undetermined significance (FLUS), follicular neoplasm, suspicious for malignancy, and malignant. Cytology and histopathology diagnoses were reviewed and confirmed by the hospital’s pathologist from Anatomical Pathology Department.

*DNA extraction and mutation analysis*

Genomic DNA from FNAB specimens was extracted using the QIAamp DNA Mini Kit (Qiagen, Hilden, Germany) according to the manufacturer’s instruction. The extracted DNA quality was measured by NanoDrop 1000 spectrophotometer (Thermo Fisher Scientific, Wilmington, DE, USA) and stored at -20^o^C. Polymerase chain reaction (PCR) was performed using HotStarTaq DNA Polymerase Kit (Qiagen, Hilden, Germany). The PCR primers were shown in Table 1. The single reaction mixture for each target (*BRAF*, *NRAS*, *TERT* promoter) in a final volume of 25 µl contained 20-50 ng of human genomic DNA template, 0.2 µM of deoxynucleoside triphosphate (dNTP) mixture, 1x PCR Buffer, 1.5 µM of MgCl_2_, 0.25 µM of each primer, and 0.25 units of HotStarTaq DNA polymerase. The amplification process was carried out with an enzyme activation step at 95^o^C for 15 minutes, followed by 35 amplification cycles (94^o^C for 30 seconds, 56^o^C for 30 seconds, and 72^o^C for 30 seconds), and a final extension at 72^o^C for 10 minutes. PCR product was evaluated by 2% agarose gel electrophoresis and visualized using Gel Doc EZ Gel Documentation System (Bio-Rad Laboratories, Hercules, CA, USA).

PCR products were purified using the QIAquick PCR Purification Kit (Qiagen, Hilden, Germany) according to the manufacturer’s protocol and continued to the sequencing process. DNA sequencing was carried out using Big Dye terminator v3.1 cycle sequencing reaction kit (Applied Biosystem, Foster City, CA, USA) with 3500 Genetic Analyzer (Applied Biosystem, Foster City, CA, USA) instrument based on the Sanger principle at our institutional sequencing facility of Dharmais Cancer Hospital. Internal negative and positive control samples were included in each testing. Mutation detection was analyzed using Snapgene Viewer software (GSL Biotech LLC, San Diego, CA, USA).

*Statistical analysis*

The patient’s clinical data were analyzed using the SPSS software version 20 for Windows (IBM Corporation, Armonk, NY, USA). Descriptive data are presented in the frequency table. Sensitivity, specificity, positive predictive value (PPV), negative predictive value (NPV) were calculated using CATmaker software version 1.1 (CEBM, Oxford, UK).

## Results

*Patient’s characteristics*

A total of 59 patients with the confirmed case were enrolled and only 50 patients were included in the final analysis. Nine patients were excluded for the following reasons: 6 patients did not undergo FNAB procedure and 3 patients had a history of thyroid surgery. As reported in Table 2, the mean age for all cases was 50.9 ± 12.0 years (range 30-75). Females were more frequent (82.0%) than males (18.0%). Forty-one (82.0%) patients had nodules symptoms between 1-5 years and 9 (18.0%) for more than 5 years. The median tumor size was 5.0 cm (range 1.0-14.0 cm). The TNM stage showed 14 (35.9%) patients in stage I, 5 (12.8%) patients in stage II, 9 (23.1%) patients in stage III, and 11 (28.2%) patients in stage IV. Sixteen (32%) patients had undergone total thyroidectomy and lymph node surgery.

All the patients included in the study underwent either preoperative or intraoperative FNAB procedures. The cytology results showed the benign in 15 (30.0%) cases, AUS/FLUS in 6 (12%) cases, follicular neoplasm in 1 (2.0%) case, suspicious for malignancy in 6 (12.0%) cases, and malignant in 12 (44.0%) cases. The cytological category of AUS/FLUS, follicular neoplasm, and suspicious of malignancy were categorized as indeterminate and found in 13 (26%) of all cases. The histopathology results showed anaplastic carcinoma (AC) in 3 (6.0%) cases, poorly differentiated thyroid carcinoma (PDTC) in 1 (2.0%) case, papillary carcinoma classic variant (PTVcv) in 23 (46.0%) cases, papillary carcinoma follicular variant (PTCfv) in 12 (24.0%) cases. The histopathological follicular adenoma (FA), thyroiditis, and goiter in a total of 11 (22.0%) patients were categorized as benign goiter. About 39 out of 50 cases (78%) were malignancies. 

When the cytological findings were confirmed by the gold standard histopathological diagnosis, it was observed that all of the 22 cytology malignant results were ultimately diagnosed to be malignant, consisting of 3 cases (13.6%) of AC, 1 case (4.5%) of PDTC, 15 cases (68.2%) of PTCcv, and 3 cases (13.6%) of PTCvf. However, 6 out of 15 benign cytology results were ultimately diagnosed as malignancy, consisting of 2 cases (13.3%) of PTCcv, and 4 cases (26.7%) of PTCvf. It means that the FNAB results have a false-negative rate of 40%. On the other hand, 11 out of 13 cases (85%) of indeterminate cytology results were diagnosed as malignant, and the rest of 2 cases (15%) were diagnosed as goiter (Table 3). 

*Mutation analysis*

**Figure 2:**
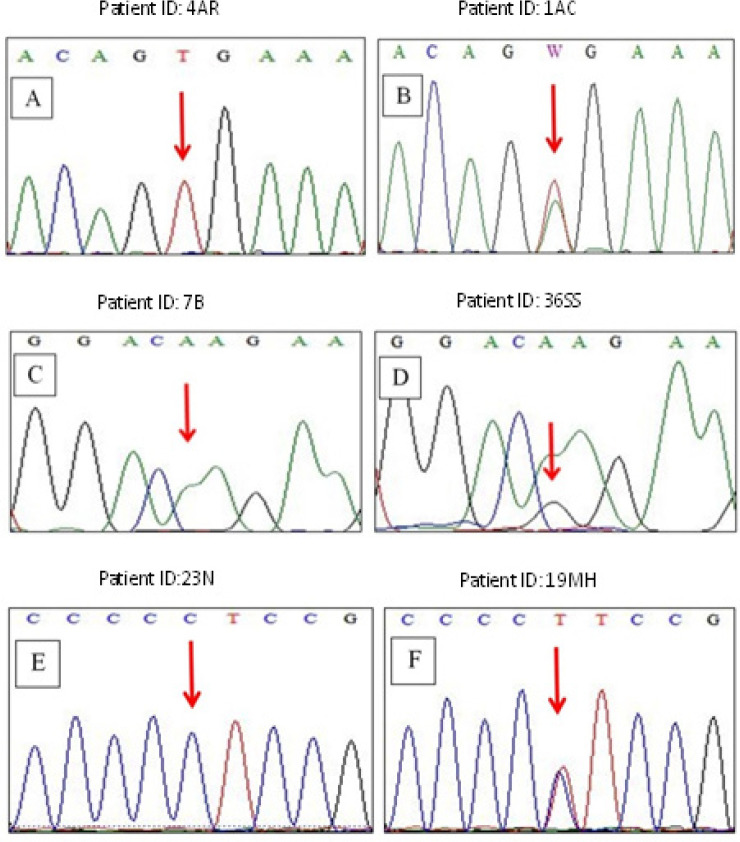
Representative Sequencing Results for *BRAF, NRAS*, and *TERT* Promoter Mutation. (A) Sequence of wildtype* BRAF *exon 15, codon 600 (GTG, arrow). (B) Sequence of mutant *BRAF* exon 15, codon 600, showing overlapping peak (GTG>GAG, arrow) indicated substitution of amino acid valine to glutamic acid. (C) Sequence of wildtype *NRAS* exon 3, codon 61 (CAA, arrow). (D) Sequence of mutant *NRAS* exon 3, codon 61, showing overlapping peak (CAA>CGA, arrow) indicated substitution of amino acid glutamine to arginine. (E) Sequence of wildtype *TERT *promoter (CTCCGG, arrow). (F) Sequence of mutant *TERT* promoter, showing overlapping peak at nucleotide base position number 1,295,228 (228C>T, arrow)

Sanger sequencing was performed to detect the hotspot mutation in *BRAF* exon 15, *NRAS* exon 3, and *TERT* promoter region in 50 FNAB specimens. The mutation analysis results of 39 malignancy cases are shown in Table 4. Our results demonstrated all *BRAF* mutation-positive cases were found in malignancy with a frequency of 31.0%. All *BRAF* mutation-positive cases were valine (V) to glutamic acid (E) substitution at codon 600 (V600E). The overlapping peak indicated the nucleotide base substitution from T to A (GTG>GAG). For *NRAS* mutation-positive cases were detected in 18.0% of malignant cases. The presence of *NRAS* mutation was mutually exclusive with *BRAF* mutation. The most common type of *NRAS* mutation was an amino acid substitution of glutamine (Q) to arginine (R) at codon 61 (Q61R). The overlapping peak indicated the nucleotide base substitution from A to G (CAA>CGA) (Figure 1.2). Following *BRAF* and *NRAS* mutation, the mutations in *TERT* promoter were found coexistence either with *BRAF* (2/5) or *NRAS* (3/5) with a total frequency of 13.0%. The only type of *TERT* mutation found in this study was nucleotide base substitution from C to T (228C>T) in the promoter region of chromosome 5. The representative traces of electropherogram results are shown in Figure 2.

**Figure 3:**
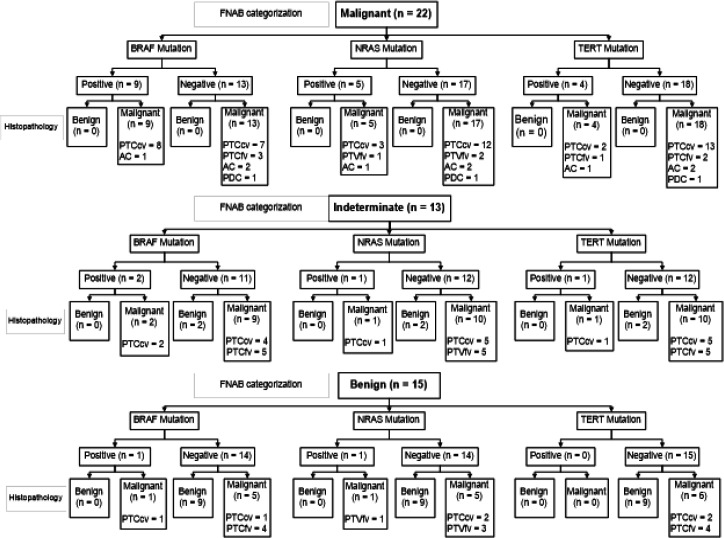
Mutation Status of FNAB Specimens. The thyroid nodules are categorized based on cytology results as malignant, indeterminate, and benign. *FNAB, fine needle aspiration biopsy; AC, anaplastic carcinoma; PDTC, poorly differentiated thyroid carcinoma; PTCcv, papillary thyroid carcinoma classic variant; PTCfv, papillary thyroid carcinoma follicular variant

Based on the cytological findings, 22 cases were categorized as malignant, 13 indeterminate, and 15 benign cases. In the malignant group, *BRAF* mutation was detected in 9 cases which ultimately proved to be all malignant histopathology results, which consisted of 8 PTCcv cases and 1 AC case. *NRAS* mutation was detected in 5 cases which consisted of 3 PTCcv cases, 1 PTCfv case, and 1 AC case. *TERT* mutation was detected in 4 cases which consisted of 2 PTCcv cases, 1 PTCfv case, and 1 AC case. In the indeterminate group, *BRAF* mutation was detected in 2 cases which ultimately proved to be malignant and was only found in PTCcv. *NRAS* mutation was detected in 1 case which proved to be PTCcv, while *TERT* mutation was detected in 1 case and proved to be PTCcv. In the benign group, *BRAF* mutation was detected only in 1 case which proved to be PTCcv. *NRAS* mutation was detected in 1 case and proved to be PTCfv, while no *TERT* mutation was detected in all benign cytology cases (Figure 3).

*Diagnostic value of FNAB and molecular testing*

To assess the diagnostic value of FNAB and each mutation, we compared the results to the histopathology diagnosis. The sensitivity, specificity, PPV, and NPV of each test are shown in Table 5. Of the 50 thyroid nodule cases, *BRAF*, *NRAS*, and *TERT* promoter mutation correctly detected thyroid cancer respectively in 12, 7, and 5 cases from 39 malignancy cases. The sensitivity resulted were 31% (95% CI, 16%-45%), 18% (95% CI, 6%-30%), and 13% (95% CI, 2%-23%), respectively. All mutations were correctly identified as negative for malignancy in all 11 benign cases, resulting in a specificity of 100% (95% CI, 100%-100%). The PPV for all mutation-positive cases of malignancy was 100% (95% CI, 100%-100%), and the NPV resulted respectively, 29% (95% CI, 15%-43%), 26% (95% CI, 13%-39%), 24% (95% CI, 12%-37%). If we add the diagnostic value of molecular testing to FNAB, it will result in a sensitivity of 69% (95% CI, 55%-84%), specificity of 100% (95% CI, 100%-100%), PPV of 100% (95% CI, 100%-100%), and NPV of 48% (95% CI, 27%-68%).

## Discussion

Thyroid cancer incidence has been reported to be the top 5 cause of cancer in Indonesia accounting for 4.2% of all new cancer cases among women in 2018 (World Health Organization, 2019). The case number of thyroid cancer is expected to continue to increase in the future years due to environmental factors such as radiation exposure, pollutants, and high iodine intake (Zimmermann and Galetti, 2015). The growing use of advanced diagnostic imaging and FNAB are also suspected of being associated with the increased case of thyroid cancer (Kitahara and Sosa, 2016). 

FNAB is notable to be the most accurate and cost-effective examination in evaluating thyroid nodules (Auger et al., 2013). In this study, the application of FNAB has a sensitivity of 56% and has a false negativity rate of 40%. This FNAB performance was relatively poor compared to the other studies which have a sensitivity ranging from 65% to 98% and a false negative rate below 5%. It might be caused most of the cases were taken intraoperatively without ultrasound guidance. Other factors including the inadequate amount of cells for the analysis, fixation method, presence of non-homogenous nodule, and subjectivity of the pathologist who interprets the cytology slides could affect the FNAB results (Bozbiyik et al., 2017). As a consequence, many patients may undergo diagnostic surgery and raising the risk of morbidity if they are ultimately diagnosed as a benign disease. Therefore, additional diagnostic tests are necessary to improve the accurate diagnosis of FNAB, especially in indeterminate cytology results. 

To date, molecular testing has been widely studied for thyroid cancer with *BRAF* mutation as the most commonly used biomarker for preoperative diagnosis using FNAB specimens (Nikiforova and Nikiforov, 2009). Furthermore, the use of *BRAF* mutation for postoperative analysis using primary tumor tissue is also important to predict the need for radioactive iodine ablation (RAI) therapy (Han et al., 2014). It was one of our limitations that we did not do mutation assessment from the primary tumor tissue and It will be a better study if we can examine it in the future. However, in our clinical setting, since investigating the mutational status for pre- and postoperative is not covered by public health insurance and is unaffordable for most of the patients, the initial mutation analysis is sufficient even for determining adjuvant RAI therapy. This is supported by our previous results that showed high concordance between preoperative *BRAF* mutation analysis from FNAB specimens and postoperative from the primary tumor (Brahma et al., 2013). 

Our previous study showed *BRAF* mutation has high specificity and PPV for PTC, and it could be used as guidance for extensive thyroidectomy in selective patients (Brahma et al., 2013). However, a low rate of sensitivity remains a problem and concludes that *BRAF* mutation alone is insufficient for routine clinical settings. Additional biomarkers including *NRAS* and *TERT* promoter mutations are acknowledged to have a potential clinical significance in thyroid cancer and could be applied in synergy with *BRAF* mutation. 

In this study, 50 FNAB specimens were analyzed for *BRAF*, *NRAS*, and *TERT* mutations by Sanger sequencing. As a simple molecular panel, this approach is more cost-effective compared to next-generation sequencing (NGS) and could easily be implemented in the clinical setting, considering Sanger sequencing that has already become a routine procedure for mutation analysis in our hospital. Despite some detection limit issues, a study by Chung et al., (2006) showed that Sanger sequencing is still reliable to detect the mutation from FNAB specimens. 

Our results demonstrated that *BRAF*, *NRAS*, and *TERT* mutations were found in the frequency of 31%, 18%, and 12%, respectively. The prevalence of these mutations in thyroid cancer showed varying numbers in different studies. A study by Decaussin-Petrucci et al., (2017) in France has shown the frequency of *BRAF*, *NRAS*, and *TERT* promoter mutations of 44.8%, 14.7%, and 5.5%, respectively. Another study by Argyropoulou et al., (2018) from the Greek population revealed the lower prevalence of these mutations in 17%, 3.4%, and 3.4%, respectively. The different ethnicity, environments, specimen types, and detection methods may affect the presence of these mutations. 

The identification of mutation confirmed that *BRAF* V600E was mainly detected in PTC with classic variant (PTCcv) (47.8%), but at variance which included AC (33.3%). In agreement with the previous study, *BRAF* mutation has been reported in 30-80% of PTC, and also in AC which develops from pre-existing PTC, but is never detected in follicular carcinoma or benign nodules (Lee et al., 2007). Meanwhile, *NRAS* represents the second most frequent mutation and found mutually exclusive with *BRAF* mutation in this study. *NRAS* mutation accounted for 18% including PTCcv (4 cases), PTCfv (2 cases), and AC (1 case). This finding was in line with other country reports ranging from 15% to 20% mostly found in follicular carcinoma or follicular variant papillary types. Moreover, it can also be found in poorly differentiated, anaplastic, and even medullary carcinomas (Nikiforova and Nikiforov, 2009; Patel et al., 2017). However, the frequency of *NRAS* mutations in this study was lower in comparison to the study from Korea which has 26.8% (Bae et al., 2014). The low number of *NRAS* mutations particularly resulted from the few cases of follicular papillary carcinoma (24%) and follicular adenoma (2%) which commonly harbor *NRAS* mutation. 

*TERT* promoter mutations were detected less common in our specimen series (13%). However, our result has a higher frequency than Liu and Xing (2014) study which only accounts for 7% (9/129 patients). *TERT* promoter mutations are generally found only in aggressive thyroid cancer, with the frequency ranging from 4.7% to 25.5% (Liu et al., 2016). Interestingly, all *TERT* mutations that appeared in this study were identified co-existence with *BRAF* (40%) and *NRAS* mutation (60%). This fact suggests that *TERT* promoter mutations can occur simultaneously due to different oncogenic signal transduction pathways (Moon et al., 2017). According to previous studies, the coexisting of *TERT* and *BRAF* mutations were known to have a synergistic effect on aggressive clinicopathological characteristics in PTC (Liu et al., 2016; Liu et al., 2012). Nevertheless, the meta-analysis study by Liu et al., (2016) concluded that the coexistence of two mutations to predict the worst outcomes in thyroid cancer is unclear and needs further research. 

Based on diagnostic utility, the addition of multiple molecular testing has significantly raised the sensitivity of FNAB cytology examination from 56% to 69%. If we compare to our previous study (*BRAF* mutation alone), it was generating only 41% of sensitivity (Brahma et al., 2013). This study proves better progress after we implement multiple molecular testing. The combination testing correctly diagnosed 27 out of 39 thyroid carcinomas, including 2 benign and 3 indeterminate cases in cytology which have *BRAF* mutation-positive. Moreover, all of the mutations-positive in thyroid nodules were detected only in malignant cases regardless of the cytological findings, which represent PTC, PTCfv, and AC. Therefore, our results confirmed 100% specificity and PPV in predicting the disease. 

We also found the improvement for FNAB cytology false-negative rate after the combination of molecular testing from 40% to 26.7%. Two out of 6 false-negative results were rescued by *BRAF* (1/6) which was ultimately diagnosed as PTCcv and *NRAS* (1/6) which was diagnosed as PTCvf. Surprisingly, one of the *NRAS* mutation-positive cases is represented among the micro PTC follicular variant, further confirming the significance of FNAB evaluation in small nodules size <1 cm. In this scenario, molecular testing should be conducted, since it is capable to detect the possible cytological false-negative results. The discrepancy in this study was higher (34%) compared to the reported studies with 15.3% (Yang et al., 2007) demonstrating a moderate accuracy (66%) of this approach. The addition of molecular testing could reduce the discrepancy rate between the preoperative examination and postoperative (histopathology) by 10%.

Despite the great improvement of FNAB diagnostic sensitivity, there are still some issues related to the molecular test ability to detect malignancy in indeterminate and benign cytology results. 11 out of 13 cases of indeterminate cytology were ultimately diagnosed as malignant but only 4 cases that harbor mutation (Table 4). One of the reasons was the high prevalence of AUS/FLUS cytology in indeterminate cases may affect the sensitivity of molecular testing. AUS/FLUS cytology and follicular neoplasms that lead to malignancy have more likely altered *HRAS *gene than *NRAS* with a frequency of up to 94% (34). In benign cytology results, there were 6 out of 15 cases which ultimately diagnosed as malignant with 2 of them were found to have *BRAF* and *NRAS* mutation preoperatively (Table 2). The most malignant cases detected from benign cytology was PTCfv. There were only 2 out of 12 (17%) PTCfv cases harboring *NRAS* mutation. This result shows the distinction from most published studies that revealed *NRAS* mutation at codon 16 to be common in PTCvf (37–39). It can be explained by Howitt et al., (2013) study that another common genetic alteration in PTCfv is HRAS mutation alongside RET/PTC1 rearrangement (Di Cristofaro et al., 2006). 

Taken together, the additional molecular testing of *BRAF*, *NRAS*, and *TERT* promoter mutations not only enabled the identification of thyroid malignancies in FNAB cytology but also allowed the prediction of the aggressive characteristics of thyroid cancer. Based on various reports, *BRAF* and *TERT* mutation are associated with the older age, female gender, bigger tumor size, extrathyroidal extension, lymph node metastasis, multifocality, advanced stage, and recurrence (Jin et al., 2018; Kebebew et al., 2007; Xing, 2005). Meanwhile, *NRAS* preferentially associated with follicular-patterned thyroid lesions, which theoretically represent precursor lesion for the malignancy (Schulten et al., 2013). Our study also demonstrated most of the patients with *BRAF*, *NRAS*, and *TERT* promoter mutation tend to undergo extensive surgery (Table 2). According to the previous investigations, the identification of *BRAF* and *TERT* promoter mutation in thyroid nodules enabled preoperative stratification of the surgical treatment, leading to total thyroidectomy and most likely prophylactic central lymph node dissection if the clinicopathological setting is appropriate (Melo et al., 2014; Paschke et al., 2017; Trimboli et al., 2016; Xing, 2013).

Indeed, it should be noted that the combination of molecular testing and FNAB was unable to detect the rest of the 31% thyroid malignancy cases in this study. Also, the high cost and the unavailability of public health insurance coverage remains a problem to perform molecular testing for patients in Indonesia. These conditions suggest the implementation of molecular testing only used for selective cases especially for patients who most likely harbor the mutations. Most investigators suggested the use of molecular testing for the following criteria: patients with high-risk nodules to reinforcing malignancy; patients with low-risk nodules or indeterminate cytology results to avoid diagnostic surgery (Danilovic and Marui, 2018). However, our results confirmed that patients with benign cytology could be included for molecular testing. It can be understood due to *BRAF*/*NRAS* mutation-positive was also detected in the benign lesion (Table 2) which was ultimately diagnosed as malignant in histopathology. A supporting study from Puzziello et al., (2016) showed benign thyroid nodules which harbor RAS mutations will grow more rapidly than the wildtype. Therefore, detecting mutations in thyroid nodules with benign cytology might be useful in deciding more appropriate surgical treatment. 

Finally, we acknowledge that the present study has several limitations. The first is concerning a small number of cases with the complete clinical data in this study leading to insufficient statistical power. Thus, we were unable to perform the analysis of the relationship between the mutational status and clinicopathology variables. Second, most of the FNAB specimens were not examined for the cell sufficiently under a microscope examination which makes this study have possible bias measurement. Third, the implementation of a molecular testing panel using limited genetic markers may not be enough to distinguish the thyroid malignancies in indeterminate or malignant cytology. Genetic alterations that are already known occurred in thyroid cancer such as, RET/PTC (20%), all-RAS mutation (10%), and TRK (<5%) that might be present in our specimens but were not evaluated by this study (Kebebew et al., 2007). The 2009 revised American Thyroid Association (ATA) management guidelines recommend using molecular markers such as *BRAF*, all-RAS, RET/PTC, PAX8/PPARγ, and Galectin-3 in cases with indeterminate cytology (Jin et al., 2018). 

In conclusion, the molecular testing of *BRAF*, *NRAS*, and *TERT* mutations can improve the sensitivity of thyroid FNAB. Detecting for the mutations might be beneficial for more definitive treatment in selective cases. However, the NPV is relatively low to avoid the need for diagnostic surgery. Therefore, further studies using a wide spectrum of biomarkers are needed to make a better indication and algorithm for molecular testing.

**Table 1:** Primer Sequence

Primer Name	Forward sequence (5’-3’)	Reverse sequence (5’-3’)	Product
*BRAF* exon 15	GACTCTAAGAGGAAAGATGAAGTAC	CACTGATTTTTGTGAATACTGGGAC	394 bp
*NRAS* exon 3	TCTTACAGAAAACAAGTGGT	GTAGAGGTTAATATCCGCAA	174 bp
*TERT* promoter	ACGAACGTGGCCAGCGGCAG	CTGGCGTCCCTGCACCCTGG	474 bp

**Table 2:** Frequency of *BRAF, NRAS*, and *TERT* Mutation According to Clinical Characteristics of Patients

Characteristics	n (%)	*BRAF* mutation		*NRAS* mutation		*TERT* mutation	
		Positive (n = 12)	Negative (n = 38)	Positive (n = 7)	Negative (n = 43)	Positive (n = 5)	Negative (n = 45)
Gender							
Male	9 (18.0)	4 (33.3)	5 (13.2)	0 (0.0)	9 (20.9)	1 (20.9)	8 (17.8)
Female	41 (82.0)	8 (66.7)	33 (86.8)	7 (100.0)	34 (82.9)	4 (79.1)	37 (82.2)
Age (years) Mean ± SD	50.9 ± 12.0						
Range (years)							
<45	18 (36.0)	5 (41.7)	13 (34.2)	2 (28.6)	16 (37.2)	0 (0.0)	18 (40.0)
≥45	32 (54.0)	7 (58.3)	27 (65.8)	5 (71.4)	27 (62.8)	5 (100.0)	27 (60.0)
Onset (years)							
<5	41 (82.0)	9 (75.0)	32 (84.2)	6 (85.7)	35 (81.4)	5 (100.0)	36 (80.0)
≥5	9 (18.0)	3 (25.0)	6 (15.8)	1 (14.3)	8 (18.6)	0 (0.0)	9 (20.0)
Tumor size (cm) Median	5.0 (1.0-14.0)						
Range (cm)							
<4	17 (34.0)	2 (16.7)	15 (39.5)	4 (57.1)	13 (30.2)	2 (40.0)	15 (33.3)
≥4	33 (66.0)	10 (83.3)	23 (60.5)	3 (42.9)	30 (69.8)	3 (60.0)	30 (66.7)
Stage (n = 39)							
I	14 (35.9)	2 (16.7)	12 (44.4)	2 (28.6)	12 (37.5)	0 (0.0)	14 (41.2)
II	5 (12.8)	2 (16.7)	3 (11.1)	1 (14.3)	4 (12.5)	1 (20.0)	4 (11.8)
III	9 (23.1)	4 (33.3)	5 (18.5)	1 (14.3)	8 (25.0)	1 (20.0)	8 (23.5)
IV	11 (28.2)	4 (33.3)	7 (26.0)	3 (42.8)	8 (25.0)	3 (60.0)	8 (23.5)
AMES risk (n = 39)							
Low risk	22 (56.4)	5 (41.7)	17 (63.0)	3 (42.9)	19 (59.4)	2 (40.0)	20 (58.8)
High risk	17 (43.6)	7 (58.3)	10 (37.0)	4 (57.1)	13 (40.6)	3 (60.0)	14 (41.2)
LN metastasis (n = 16)							
Negative	3 (18.3)	1 (14.3)	2 (22.2)	2 (50.0)	1 (8.3)	2 (50.0)	1 (8.3)
Positive	13 (81.3)	6 (85.7)	7 (77.8)	2 (50.0)	11 (91.7)	2 (50.0)	11 (91.7)
Surgery							
Lobectomy	13 (26.0)	0 (0.0)	13 (34.2)	0 (0.0)	13 (30.2)	0 (0.0)	13 (29.0)
ET	2 (4.0)	0 (0.0)	2 (5.3)	0 (0.0)	2 (4.7)	0 (0.0)	2 (4.4)
TT	13 (26.0)	3 (25.0)	10 (26.3)	2 (28.6)	12 (27.9)	1 (20.0)	14 (31.1)
TT + LN	16 (32.0)	7 (58.4)	9 (23.7)	4 (57.1)	11 (25.6)	3 (60.0)	11 (24.4)
Others	6 (12.0)	2 (16.6)	4 (10.5)	1 (14.3)	5 (11.6)	1 (20.0)	5 (11.1)
Cytology							
Benign	15 (30.0)	1 (8.3)	14 (36.8)	1 (14.3)	14 (32.6)	0 (0.0)	15 (33.3)
AUS/FLUS	6 (12.0)	0 (0.0)	6 (15.8)	0 (0.0)	6 (14.0)	0 (0.0)	6 (13.3)
FN	1 (2.0)	0 (0.0)	1 (2.6)	0 (0.0)	1 (2.3)	0 (0.0)	1 (2.2)
SFM	6 (12.0)	2 (16.7)	4 (10.5)	1 (14.3)	5 (11.6)	1 (20.0)	5 (11.1)
Malignant	22 (44.0)	9 (75.0)	13 (34.2)	5 (71.4)	17 (39.5)	4 (80.0)	18 (40.0)
Histopathology							
AC	3 (6.0)	1 (8.3)	2 (5.3)	1 (14.3)	2 (4.7)	1 (20.0)	2 (4.4)
PDTC	1 (2.0)	0 (0.0)	1 (2.6)	0 (0.0)	1 (2.3)	0 (0.0)	1 (2.2)
PTCcv	23 (46.0)	11 (91.7)	12 (31.6)	4 (57.1)	19 (44.2)	3 (60.0)	20 (44.4)
PTCfv	12 (24.0)	0 (0.0)	12 (31.6)	2 (28.6)	10 (23.3)	1 (20.0)	11 (24.5)
Benign goiter	11 (22.0)	0 (0.0)	11 (28.9)	0 (0.0)	11 (25.5)	0 (0.0)	11 (24.5)
Mutation frequency (n = 39)		12 (31.0)		7 (18.0)		5 (12.0)	

*AC, anaplastic carcinoma; PDTC, poorly differentiated thyroid carcinoma; PTCcv, papillary thyroid carcinoma classic variant; PTCfv, papillary thyroid carcinoma follicular variant; FN, follicular neoplasm; SFM, suspicious for malignancy; ET, endoscopic thyroidectomy; TT, total thyroidectomy; TT + LN, total thyroidectomy + lymph node surgery.

**Table 3:** Cytology *FNAB* Findings on Histopathology Results

Cytology	Histopathology				
	Benign goiter	PTCcv	PTCfv	PDTC	AC
Benign (n = 15)	9 (60.0)	2 (13.3)	4 (26.7)	0 (0.0)	0 (0.0)
AUS/FLUS (n = 6)	2 (33.3)	2 (33.3)	2 (33.3)	0 (0.0)	0 (0.0)
FN (n = 1)	0 (0.0)	1 (100.0)	0 (0.0)	0 (0.0)	0 (0.0)
SFM (n = 6)	0 (0.0)	3 (50.0)	3 (50.0)	0 (0.0)	0 (0.0)
Malignant (n = 22)	0 (0.0)	15 (68.2)	3 (13.6)	1 (4.5)	3 (13.6)

*FN, follicular neoplasm; SFM, suspicious for malignancy; AC, anaplastic carcinoma; PDTC, poorly differentiated thyroid carcinoma; PTCcv, papillary thyroid carcinoma classic variant; PTCfv, papillary thyroid carcinoma follicular variant

**Table 4:** Mutation Analysis Results of 50 Thyroid Nodule Cases

Case ID	Mutation	Cytology	Histology	Case ID	Mutation	Cytology	Histology
1AC	*BRAF* V600E	Malignant	PTCcv	26RJ	-	Malignant	PTCcv
2AD	-	AUS/FLUS	PTCfv	27R	*BRAF* V600E	Malignant	PTCcv
3AP	-	SFM	mPTCfv	28R	*NRAS* Q61R	Malignant	PTCcv
4AR	-	Benign	FA	29R	*BRAF* V600E	SFM	PTCcv
5B	-	FN	mPTCcv	30S	-	AUS/FLUS	PTCfv
6BI	-	AUS/FLUS	Goiter	31S	-	Benign	Goiter
7B	-	Benign	mPTCfv	32S	-	Benign	PTCcv
8C	-	Malignant	PTCcv	33SJ	-	Benign	PTCfv
9D	-	Benign	Goiter	34S	-	Malignant	PTCfv
10ES	*NRAS* Q61R, TERT 228C>T	Malignant	PTCfv	35S	-	Benign	Goiter
11H	-	AUS/FLUS	PTCcv	36SS	*NRAS* Q61R, TERT 228C>T	Malignant	PTCcv
12J	*BRAF* V600E	Malignant	PTCcv	37SM	-	Malignant	AC
13JK	*NRAS* Q61R	SFM	PTCcv	38SU	-	AUS/FLUS	PTCcv
14KD	-	Benign	Thyroiditis	39S	-	Malignant	PDTC
15LL	*NRAS* Q61R, TERT 228C>T	Malignant	AC	40SI	-	Malignant	PTCfv
16M	-	Malignant	PTCcv	41T	*BRAF* V600E	Malignant	AC
17M	*BRAF* V600E	Malignant	PTCcv	42TR	*NRAS *Q61R	Benign	mPTCfv
18MH	-	Benign	Goiter	43TT	*NRAS* Q61R	Malignant	PTCcv
19MH	*BRAF* V600E, TERT 228c>T	Malignant	PTCcv	44TD	*BRAF *V600E	Malignant	PTCcv
20MI	-	Malignant	PTCcv	45T	-	Malignant	PTCcv
21NS	-	SFM	PTCfv	46TN	-	Benign	Goiter
22N	*BRAF* V600E	Benign	PTCcv	47T	-	Benign	mPTCfv
23N	-	Benign	Goiter	48T	-	Benign	Goiter
24N	-	SFM	PTCfv	49Y	*BRAF* V600E, TERT 228C>T	SFM	PTCcv
25RE	*BRAF* V600E	Malignant	PTCcv	50Y	-	AUS/FLUS	Goiter

*AC, anaplastic carcinoma; PDTC, poorly differentiated thyroid carcinoma; PTCcv, papillary thyroid carcinoma classic variant; PTCfv, papillary thyroid carcinoma follicular variant; FA, follicular adenoma; FN, follicular neoplasm; SFM, suspicious for malignancy

**Table 5:** Diagnostic Value of *FNAB* and Molecular Testing in 50 Thyroid Nodule Patients

Test	SN (95% CI)	SP (95% CI)	PPV (95% CI)	NPV (95% CI)	Accuracy (95% CI)
*FNAB*	56 % (16-45)	100 % (100-100)	100 % (100-100)	39 % (21-57)	66% (51-79)
*BRAF*	31 % (16-45)	100 % (100-100)	100 % (100-100)	29 % (15-43)	46 % (32-61)
*NRAS*	18 % (6-30)	100 % (100-100)	100 % (100-100)	26 % (13-39)	36 % (23-51)
*TERT*	13 % (2-23)	100 % (100-100)	100 % (100-100)	24 % (12-37)	32 % (20-47)
*BRAF + TERT + NRAS*	49 % (33-64)	100 % (100-100)	100 % (100-100)	35 % (19-52)	60 % (45-74)
*FNAB + BRAF + TERT + NRAS*	69 % (55-84)	100 % (100-100)	100 % (100-100)	48 % (27-68)	76 % (62-87)

*SN, sensitivity; SP, specificity; PPV, positive predictive value; NPV, negative predictive value
